# Supplementary material for: The impact of global warming on the signature virulence gene, thermolabile hemolysin, of Vibrio parahaemolyticus
Source: Microbiol Spectr. 2023 Oct 16;11(6):e01502-23. doi: 10.1128/spectrum.01502-23 (PMC10715048; doi:10.1128/spectrum.01502-23)
Supplement: Supplemental figures and tables — Fig. S1 and S2; Tables S1 to S5. [file spectrum.01502-23-s0003.pdf]

Manuscript ID: Spectrum01502-23

Supplementary Information For

The impact of global warming on the signature virulence gene, thermolabile hemolysin, of *Vibrio parahaemolyticus*

Weishan Zhang [a, b†], Keyu Chen [a, b†], Lin Zhang [c], Ximeng Zhang [d], Baoli Zhu [a, b], Na Lv [a#], Kaixia Mi [a, b #]

[a] CAS Key Laboratory of Pathogen Microbiology and Immunology, Institute of Microbiology, Chinese Academy of Sciences, Beijing, China.

[b] Savaid Medical School, University of Chinese Academy of Sciences, Beijing, China.

[c] Shijiazhuang Customs Technology Center, Hebei, China.

[d] Science and Technology Research Center of China Customs, Beijing, China.

[†] These authors contributed equally to this work.

[#] Correspondence to Kaixia Mi, mik@im.ac.cn, Na Lv, lvna@im.ac.cn.

Present address: NO.1 Beichen West Road, Chaoyang District, Beijing 100101, China.

This PDF file includes:

Figure supplement 1

Figure supplement 2

Table supplement 1

Table supplement 2

Table supplement 3

Table supplement 4

Figure supplement 1

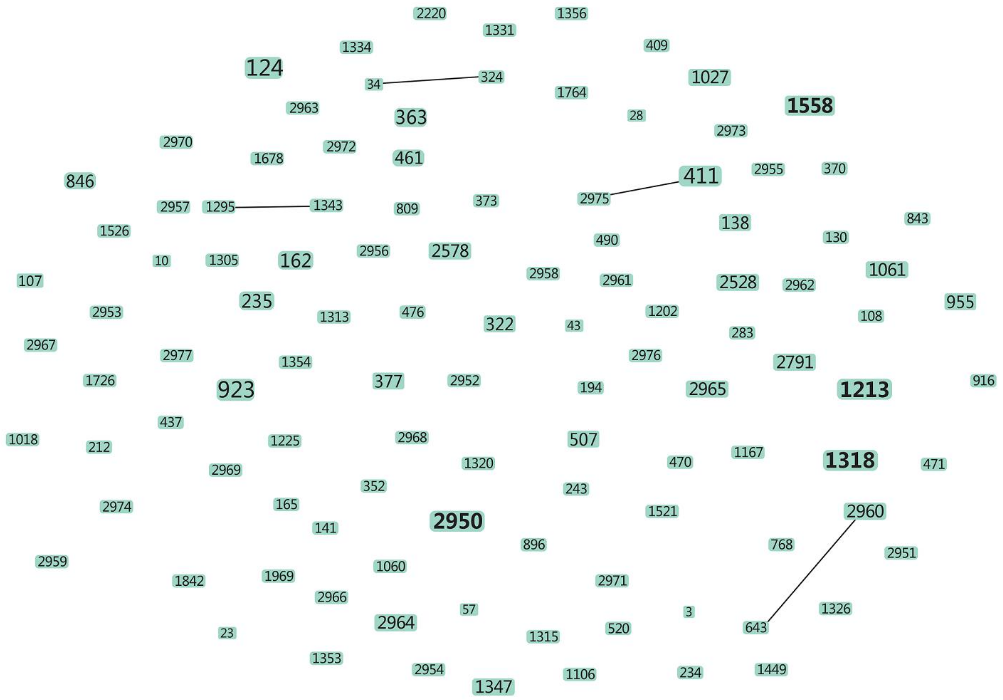

Fig.S1 The clonal complex analysis of *V. parahaemolyticus* strains using goeBURST (v1.2.1). Two STs are considered single-locus variants (SLV) when they differ from each other at a single locus. Double-locus variants (DLVs) are any two STs that differ in the two loci. The aqua line indicates connected DLV STs.

Figure supplement 2

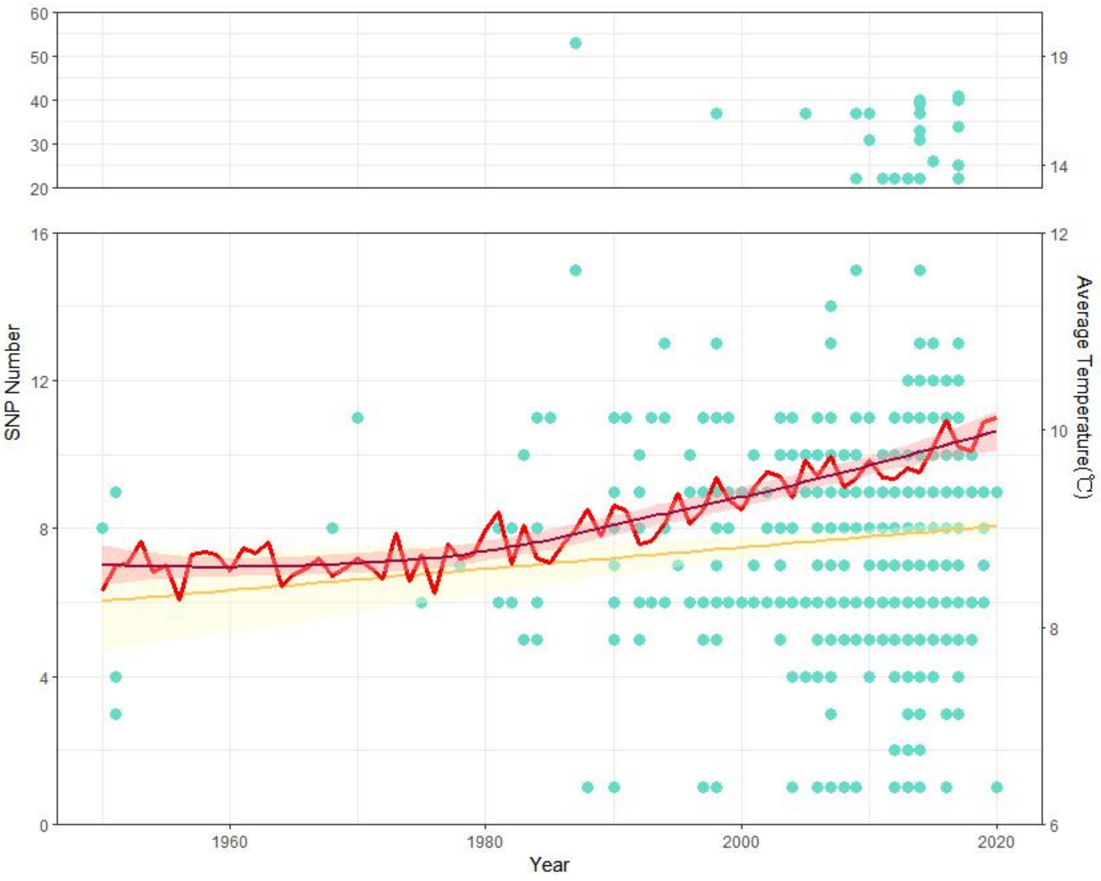

Fig.S2 The *tlh* mutated with global temperatures increased. The red line indicated the global average temperature and its fitting curve. The light point indicated each strain's SNP number of *tlh* in different years and the orange line was its fitting curve. The red and the light orange shadows indicated the 99% confidence interval.

Table S1 ANI analysis of 245 *V. parahaemolyticus* strains.

Strain	ANI (%)
vp.301	96.70
vp.302	96.73
vp.303	96.55
vp.304	96.66
vp.305	96.76
vp.306	96.53
vp.307	96.21
vp.308	96.73
vp.310	96.78
vp.311	96.68
vp.312	97.10
vp.313	96.52
vp.314	96.70
vp.315	96.67
vp.317	96.97
vp.318	97.19
vp.319	96.48
vp.320	96.89
vp.321	96.80
vp.322	96.79
vp.323	97.05
vp.324	96.69
vp.325	96.25
vp.326	96.39
vp.327	96.73
vp.328	96.62
vp.330	96.54
vp.331	96.84
vp.332	97.81
vp.333	96.20
vp.334	97.06
vp.335	97.16
vp.336	96.53
vp.337	96.87
vp.338	96.76
vp.339	96.79
vp.340	96.70
vp.341	85.77
vp.342	96.59

vp.344	97.00
vp.345	96.86
vp.347	96.64
vp.348	96.67
vp.349	96.92
vp.350	96.65
vp.351	96.85
vp.352	96.80
vp.353	96.78
vp.354	96.82
vp.355	96.90
vp.356	96.75
vp.357	96.99
vp.358	96.68
vp.359	84.09
vp.360	96.95
vp.362	96.53
vp.363	96.75
vp.364	96.97
vp.366	96.63
vp.367	97.13
vp.368	96.72
vp.369	96.70
vp.371	96.46
vp.372	96.52
vp.374	97.01
vp.375	97.01
vp.376	96.76
vp.377	96.75
vp.378	96.81
vp.380	96.49
vp.381	96.96
vp.382	96.82
vp.383	96.80
vp.384	96.55
vp.385	97.19
vp.386	97.33
vp.388	85.81
vp.390	96.72
vp.391	96.86
vp.392	97.03
vp.393	97.09

vp.394	96.99
vp.395	96.38
vp.396	96.97
vp.397	96.69
vp.398	96.80
vp.399	96.77
vp.400	97.22
vp.401	96.69
vp.402	96.62
vp.403	98.71
vp.404	97.14
vp.405	96.56
vp.406	96.61
vp.407	83.90
vp.408	96.46
vp.409	96.49
vp.410	96.52
vp.411	96.74
vp.412	96.88
vp.413	96.80
vp.414	96.85
vp.415	96.77
vp.416	96.59
vp.417	96.74
vp.418	96.64
vp.419	96.37
vp.420	96.80
vp.421	96.87
vp.423	96.94
vp.424	96.80
vp.425	97.96
vp.426	96.80
vp.427	96.77
vp.428	96.85
vp.429	96.92
vp.430	96.54
vp.431	96.96
vp.432	96.86
vp.433	96.42
vp.435	96.72
vp.437	96.70
vp.438	96.65

vp.439	96.83
vp.441	96.58
vp.442	96.75
vp.443	96.56
vp.444	96.61
vp.445	96.78
vp.446	96.99
vp.448	96.73
vp.449	96.61
vp.450	96.69
vp.451	96.39
vp.452	96.67
vp.453	96.87
vp.454	96.77
vp.455	96.74
vp.456	96.80
vp.458	96.81
vp.459	96.73
vp.460	96.78
vp.462	96.56
vp.467	96.74
vp.468	96.81
vp.469	96.61
vp.470	96.76
vp.471	96.80
vp.472	96.39
vp.473	96.81
vp.474	96.85
vp.475	97.02
vp.476	96.72
vp.477	96.86
vp.478	96.64
vp.481	97.03
vp.482	96.72
vp.483	96.74
vp.484	96.78
vp.485	96.70
vp.486	96.29
vp.487	96.75
vp.489	96.89
vp.490	96.68
vp.491	96.66

vp.492	96.57
vp.493	96.49
vp.496	96.39
vp.498	96.84
vp.499	96.87
vp.500	96.60
vp.501	96.91
vp.502	96.55
vp.503	96.83
vp.504	96.76
vp.505	95.84
vp.506	96.79
vp.507	96.91
vp.508	96.77
vp.509	96.51
vp.510	96.89
vp.511	96.49
vp.512	96.53
vp.513	96.92
vp.514	96.49
vp.515	97.03
vp.524	97.07
vp.525	96.84
vp.526	96.94
vp.527	96.75
vp.528	96.74
vp.533	96.77
vp.535	96.69
vp.536	96.61
vp.537	96.84
vp.538	96.74
vp.539	96.67
vp.541	97.11
vp.551	96.98
vp.553	96.88
vp.554	96.52
vp.555	96.63
vp.557	96.66
vp.558	96.94
vp.560	96.79
vp.561	96.69
vp.562	97.10

Table S2 General genomic features and annotation of 241 *V. parahaemolyticus* strains collected from China Customs.

Strains	Source	Location	Date of isolation	ST	Number of reads	Reads length	Genome length	No.of contings	GC%	N50	L50	Sequencing Depth	Gene	CDS	tRNA	tmRNA	rRNA	tdh/trh
vp.403	Dungeness Crab	Canada	2007/9/14	3	4561263	150	5025936	59	45.39	372260	6	204×	4601	4510	86	1	4	-/-
vp.375	Dungeness Crab	the United States	2006/7/11	10	8795678	150	5207587	51	45.19	481064	4	197×	4800	4707	88	1	4	-/-
vp.395	Dungeness Crab	Canada	2006/8/25	23	4805523	150	4930783	63	45.31	194837	8	208×	4522	4426	92	1	3	+/+
vp.335	Panopea Generosa	the United States	2006/8/25	28	5251416	150	5060826	48	45.28	255154	6	202×	4617	4516	96	1	4	-/-
vp.384	Panopea Generosa	Canada	2006/8/3	34	5316060	150	5375460	54	44.87	397862	6	190×	5079	4982	92	1	4	+/+
vp.371	Panopea Generosa	Canada	2006/7/11	43	5413222	150	5931120	96	44.01	290209	8	173×	5774	5614	155	2	3	+/+
vp.347	Panopea Generosa	the United States	2006/8/9	57	3663173	150	5102361	78	45.27	126046	12	201×	4678	4599	75	1	3	-/-
vp.430	Dungeness Crab	the United States	2007/10/26	107	7336470	150	5054477	40	45.26	450124	4	203×	4665	4578	83	1	3	-/-
vp.490	Ranina Ranina	Thailand	2008/6/11	124	4501030	150	4977397	46	45.31	410994	5	206×	4574	4485	85	1	3	-/-
vp.491	Pomfret	China	2008/6/13	124	5417274	150	4976928	48	45.31	411123	5	206×	4567	4485	78	1	3	-/-
vp.606	Whe	China	2009/10/20	124	3557746	150	5010006	93	45.24	104341	15	204×	4615	4538	73	1	3	-/-
vp.512	Clam	China	2008/8/8	130	8027992	150	6066434	50	44.03	470979	5	169×	5946	5807	133	2	4	-/+
vp.339	Dungeness Crab	the United States	2006/8/3	138	4479908	150	5230297	56	45.17	460066	5	196×	4858	4771	83	1	3	-/-

vp.377	Salmo Salar	Norway	2006/7/11	138	4483216	150	5148751	87	45.24	126688	12	199×	4736	4644	88	1	3	-/-
vp.337	Grouper	China	2006/7/11	141	4316993	150	6037390	62	44.01	409486	6	170×	5832	5703	124	2	3	-/-
vp.342	Panopea Generosa	the United States	2006/7/11	162	3608900	150	4980148	62	45.35	168665	9	206×	4529	4454	70	1	4	-/-
vp.484	Oyster	New Zealand	2008/5/15	162	3479413	150	4976751	33	45.34	411574	5	206×	4563	4471	87	1	4	-/-
vp.485	Salmo Salar	Norway	2008/5/15	162	4357313	150	4987547	39	45.33	404161	5	205×	4565	4483	77	1	4	-/-
vp.443	Croaker	China	2007/11/17	165	6911214	150	5097816	51	45.28	545738	4	201×	4673	4573	96	1	3	-/-
vp.455	Frozen Hairtail	China	2008/1/25	194	3543500	150	5014653	58	45.33	178418	10	204×	4561	4472	84	1	4	-/-
vp.601	Acharax	China	2009/8/17	212	6884308	150	5108671	44	45.15	522026	4	200×	4670	4580	85	1	4	-/-
vp.474	Salmo Salar	Norway	2008/5/8	234	7781438	150	5196823	65	45.25	364767	6	197×	4784	4690	90	1	3	-/-
vp.394	Panopea Generosa	Canada	2006/8/25	235	9305568	150	4928381	47	45.41	455909	5	208×	4510	4418	87	1	4	-/-
vp.467	Ranina Ranina	Thailand	2008/4/11	243	6383348	150	5145117	76	45.29	576249	4	199×	4800	4716	80	1	3	-/-
vp.629	Abalone	China	2010/7/15	283	3807160	150	4938353	72	45.4	123466	12	207×	4522	4438	79	1	4	-/-
vp.400	Panopea Generosa	Canada	2007/9/14	322	4173586	150	5223638	80	45.18	137149	12	196×	4786	4688	93	1	4	-/-
vp.526	Acharax	China	2008/9/23	322	6262614	150	5197629	49	45.18	395219	5	197×	4760	4666	89	1	4	-/-
vp.528	Oyster	New Zealand	2008/9/23	324	3691883	150	5131214	83	45.11	122847	12	200×	4737	4642	91	1	3	-/+
vp.330	Oratosquilla Oratoria	Burma	2007/4/29	352	8000570	150	4917535	36	45.4	473888	4	208×	4484	4396	83	1	4	-/-
vp.563	Oyster	Ireland	2008/11/4	363	4256826	150	5082476	48	45.24	401763	6	201×	4631	4533	94	1	3	-/-
vp.487	Leucothoidae	Ecuador	2008/6/11	370	3661303	150	5076655	92	45.21	129872	11	202×	4634	4543	86	1	4	-/-
vp.620	Salmo Salar	Australia	2010/7/2	373	7661092	150	4981619	74	45.37	315610	6	206×	4539	4445	90	1	3	-/-
vp.476	Oyster	New Zealand	2008/5/8	377	3923663	150	5124447	83	45.26	140566	13	200×	4674	4590	79	1	4	-/-
vp.478	Panopea Generosa	Canada	2008/5/13	377	9173924	150	5121243	52	45.27	382485	6	200×	4677	4593	79	1	4	-/-
vp.314	Scylla	Thailand	2006/12/11	409	4187866	150	5953047	114	44.16	100560	18	172×	5736	5600	130	2	4	-/-
vp.453	Frozen Hairtail	China	2008/1/25	411	7210812	150	5273140	98	45.23	148813	10	194×	4881	4797	80	1	3	-/-
vp.462	Mandarin Fish	China	2008/4/11	411	3226682	150	5152640	76	45.18	653368	3	199×	4815	4729	81	1	4	-/-

vp.536	Pomfret	China	2008/10/7	411	7472318	150	5127014	55	45.2	564819	3	200×	4776	4694	77	1	4	-/-
vp.376	Panopea Generosa	Canada	2006/7/11	437	7825084	150	5019047	48	45.28	562351	3	204×	4636	4538	93	1	4	-/-
vp.382	Gadus	Iceland	2006/7/17	461	6318950	150	5040989	42	45.23	694323	2	203×	4613	4515	94	1	3	-/-
vp.344	Salmo Salar	Norway	2006/6/27	470	4412603	150	6074643	86	44.07	215680	9	169×	5897	5768	122	2	5	-/-
vp.472	Sturgeon	China	2008/4/11	476	3977546	150	5088346	66	45.22	376574	5	201×	4690	4595	91	1	3	-/-
vp.503	Salmo Salar	Norway	2008/7/26	507	7726082	150	5062417	40	45.3	461397	5	202×	4639	4563	71	1	4	-/-
vp.504	Scylla	Burma	2008/7/26	507	7853528	150	5889667	52	44.11	402048	6	174×	5701	5582	113	2	4	-/-
vp.450	Scad	China	2008/1/25	520	4473796	150	4914053	58	45.41	402964	5	208×	4498	4400	92	1	5	-/-
vp.459	Gayu	China	2008/4/11	643	8286088	150	5085808	35	45.18	422595	4	201×	4694	4616	74	1	3	-/-
vp.573	Salmo Salar	Norway	2009/3/23	809	4383296	150	5063042	54	45.3	366187	6	202×	4632	4538	90	1	3	-/-
vp.327	Crab	China	2007/4/29	843	3893566	150	5069591	85	45.3	145058	10	202×	4672	4595	73	1	3	-/-
vp.456	Frozen Cuttle Fish	China	2008/1/25	846	5322556	150	4948698	49	45.34	193791	8	207×	4479	4402	73	1	3	-/-
vp.353	Salmo Salar	Canada	2006/8/18	896	11211980	150	5081297	65	45.25	411801	4	202×	4644	4559	81	1	3	-/-
vp.537	Lobster	Australia	2008/10/7	916	7606010	150	4928292	50	45.25	418872	5	208×	4509	4429	76	1	3	-/-
vp.435	Cray	China	2007/11/17	923	3781700	150	4853576	75	45.44	122325	12	211×	4439	4348	87	1	3	-/-
vp.437	Abalone	Australia	2007/11/17	923	4138550	150	4920539	70	45.39	178642	10	208×	4497	4412	81	1	3	-/-
vp.438	Oyster	France	2007/11/17	923	5343016	150	4916816	35	45.4	547495	3	208×	4501	4415	82	1	3	-/-
vp.439	Oyster	France	2007/11/17	923	6593448	150	4939137	42	45.38	547489	3	207×	4527	4439	84	1	3	-/-
vp.444	Salmon Fillet	China	2007/11/27	923	8316502	150	4939268	41	45.38	547489	3	207×	4534	4444	86	1	3	-/-
vp.560	Black Crab	Burma	2008/10/22	923	6784842	150	4930469	41	45.39	547489	3	208×	4514	4432	78	1	3	-/-
vp.441	Lobster	New Zealand	2007/11/17	955	6100692	150	5055023	32	45.26	518045	4	203×	4606	4518	83	1	4	-/-
vp.555	Salmo Salar	Norway	2008/10/22	1018	4961490	150	4987274	46	45.37	234473	7	205×	4563	4468	90	1	4	-/-
vp.334	Oratosquilla Oratoria	China	2006/11/12	1027	6904510	150	4903927	30	45.44	480794	4	209×	4471	4383	84	1	3	-/-
vp.481	Black Crab	Burma	2008/5/13	1027	3943130	150	4745179	86	45.48	115021	15	216×	4308	4219	85	1	3	-/-
vp.525	Clam	China	2008/9/23	1060	8774710	150	5296617	77	45.02	375905	4	193×	4892	4814	73	1	4	-/-

vp.420	Cray	China	2007/10/15	1061	8604066	150	5153501	82	45.29	168053	8	199×	4785	4684	96	1	4	-/-
vp.427	Cray	China	2007/10/15	1061	4797890	150	5092805	73	45.33	168053	6	201×	4707	4611	91	1	4	-/-
vp.386	Pseudocarcinus Gigas	Australia	2006/8/14	1106	5136206	150	4967333	43	45.35	403643	5	206×	4560	4464	91	1	4	-/-
vp.350	Abalone	China	2006/7/11	1167	7927084	150	4961395	45	45.38	374117	4	206×	4538	4456	78	1	3	-/-
vp.358	Leucothoidae	China	2006/7/4	1202	7327206	150	5073703	60	45.37	323006	6	202×	4650	4564	82	1	3	-/-
vp.331	Grouper	Thailand	2007/4/29	1213	6203338	150	6034213	74	44.01	406837	5	170×	5838	5726	107	2	3	-/-
vp.340	Oratosquilla Oratoria	China	2006/7/17	1213	4608430	150	6068847	79	43.98	313042	7	169×	5862	5732	125	2	3	-/-
vp.349	Lobster	the United States	2006/7/11	1213	4288043	150	6035378	120	44.01	119970	16	170×	5854	5716	133	2	3	-/-
vp.356	Abalone	China	2006/7/11	1213	3716133	150	6544186	537	46.51	83898	23	156×	6133	6010	116	1	6	-/-
vp.378	Lobster	Canada	2006/7/11	1213	5019640	150	5895358	96	44.13	131910	13	174×	5717	5583	129	2	3	-/-
vp.381	Salmo Salar	Norway	2006/7/17	1213	4308253	150	6023519	107	43.95	123524	16	170×	5838	5706	127	2	3	-/-
vp.574	Salmo Salar	Norway	2009/3/23	1225	7823458	150	5266263	89	45.37	190682	10	194×	4912	4819	88	1	4	-/-
vp.592	Pandalus	Canada	2009/8/12	1256	4656606	150	4878618	64	45.31	313293	6	210×	4512	4424	83	1	4	-/-
vp.305	Cray	China	2006/12/11	1295	6440066	150	5903050	67	44.08	409683	5	173×	5717	5592	119	2	4	-/-
vp.558	Scylla	Burma	2008/10/22	1305	4154826	150	4964697	80	45.34	120844	14	206×	4513	4435	74	1	3	-/-
vp.577	Panopea Generosa	the United States	2009/4/23	1313	6324050	150	5019630	53	45.29	310856	6	204×	4594	4513	76	1	4	-/-
vp.579	Salmo Salar	Norway	2009/5/19	1315	8589022	150	5024657	43	45.33	350867	6	204×	4616	4537	74	1	4	-/-
vp.583	Wild Dalian Abalone	China	2009/5/19	1318	3809416	150	6223035	80	43.97	311469	7	165×	6037	5902	130	2	3	-/-
vp.585	Black Crab	Burma	2009/5/19	1320	6918808	150	5096009	45	45.3	401932	5	201×	4683	4588	90	1	4	-/-
vp.602	Shrimp	China	2009/8/20	1326	6935264	150	4986700	41	45.27	416017	4	205×	4542	4468	70	1	3	-/-
vp.622	Tylosurus	China	2010/7/15	1331	7731010	150	5148759	56	45.34	427973	5	199×	4752	4650	97	1	4	-/-

vp.625	Melanotus Platycephalus Indicus	China	2010/7/15	1334	5307396	150	5093888	75	45.34	181388	9	201×	4721	4612	104	1	4	-/-
vp.640	Bass	China	2010/7/15	1343	5153226	150	4996239	59	45.34	373379	4	205×	4589	4507	77	1	4	-/-
vp.613	Prawns	China	2010/4/1	1347	8174970	150	5123780	88	45.23	238965	7	200×	4727	4635	87	1	4	-/-
vp.541	Oyster	New Zealand	2008/10/7	1353	4595390	150	5061395	72	45.47	290960	7	202×	4689	4601	84	1	3	-/-
vp.538	Salmo Salar	Norway	2008/10/7	1354	6994874	150	5370544	91	45.13	404265	5	191×	4937	4850	82	1	4	-/-
vp.367	Cuttlefish	China	2006/7/4	1449	7721762	150	5216339	51	45.32	491641	4	196×	4828	4735	89	1	3	-/-
vp.345	Grouper	China	2005/12/21	1521	6552932	150	6182066	60	44.02	329801	7	166×	6008	5875	128	2	3	-/-
vp.412	Dungeness Crab	Canada	2007/9/20	1526	7858538	150	5083477	40	45.21	400578	4	201×	4665	4566	94	1	4	-/-
vp.385	Crab	China	2006/8/3	1558	7084694	150	5131564	32	45.21	549754	4	200×	4703	4607	91	1	4	-/-
vp.431	Black Crab	Pakistan	2007/10/26	1558	6734500	150	5184151	54	45.16	549754	4	198×	4764	4665	94	1	4	-/-
vp.432	Brown Crab	UK	2007/10/26	1558	3683226	150	5085073	61	45.33	207630	8	201×	4650	4556	89	1	4	-/-
vp.306	Cray	China	2006/12/11	1678	6342600	150	6004116	72	43.93	369021	5	171×	5866	5731	128	2	5	-/-
vp.510	Salmo Salar	Chile	2008/7/30	1726	4902186	150	5721479	84	44.11	208651	8	179×	5617	5466	145	2	4	-/-
vp.301	Salmo Salar	Canada	2006/12/27	-	4630690	150	5056276	55	45.32	382641	5	203×	4639	4548	87	1	3	-/-
vp.302	Bass	China	2006/12/30	-	3338500	150	5170075	273	45.35	52066	32	198×	4743	4652	86	1	4	-/-
vp.303	Scylla	the Philippines	2007/1/12	-	6101852	150	5218281	58	45.36	390592	5	196×	4867	4772	90	1	4	-/-
vp.304	Cray	China	2007/1/12	-	3599123	150	4937745	80	45.36	157021	10	207×	4528	4441	82	1	4	-/-
vp.307	Oratosquilla Oratoria	China	2006/12/1	-	3965046	150	5868815	76	44.1	451234	5	174×	5746	5606	134	2	4	-/-
vp.308	Black Crab	Thailand	2006/12/1	-	8988714	150	5131558	62	45.24	484819	3	200×	4738	4642	92	1	3	-/-
vp.310	Scylla	Sri Lanka	2006/12/1	-	4866026	150	4994116	71	45.36	285707	7	205×	4583	4499	79	1	4	-/-
vp.311	Cancer Pagurus	Netherlands	2006/12/1	-	8399446	150	5350154	98	45.23	271200	6	191×	4957	4864	89	1	3	-/-
vp.312	Scylla	Thailand	2006/12/11	-	4424096	150	5139667	51	45.3	325220	5	199×	4711	4628	78	1	4	-/-

vp.313	Scylla	Thailand	2006/12/11	-	8404822	150	5176419	88	45.16	380057	4	198×	4855	4757	93	1	4	-/-
vp.315	Hairy Crab	China	2006/12/11	-	4467884	150	5242644	57	45.42	400764	4	195×	4865	4783	77	1	4	-/-
vp.317	Philippine Grouper	the Philippines	2007/4/29	-	4680740	150	4932416	30	45.47	459349	4	208×	4519	4429	86	1	3	-/-
vp.318	Dungeness Crab	Canada	2006/12/11	-	5226040	150	5122545	48	45.19	298583	5	200×	4675	4585	85	1	4	-/-
vp.319	Cray	China	2006/12/11	-	5002093	150	5131006	68	45.21	168421	9	200×	4640	4550	86	1	3	-/-
vp.320	Cray	China	2006/12/19	-	5140736	150	4922156	32	45.4	515467	4	208×	4500	4403	92	1	4	-/-
vp.321	Scylla	Sri Lanka	2006/12/19	-	8104016	150	5032970	42	45.37	476977	4	203×	4625	4533	87	1	4	-/-
vp.322	Scylla	Sri Lanka	2006/12/11	-	6400274	150	5312938	99	45.25	322607	7	193×	4880	4786	90	1	3	-/-
vp.323	Crab	Sri Lanka	2006/12/11	-	7244498	150	5655471	54	44.67	393682	5	181×	5363	5252	105	2	4	-/-
vp.324	Fiddler Crab	Bangladesh	2007/4/2	-	3990526	150	5002813	42	45.31	397196	5	205×	4582	4488	90	1	3	-/-
vp.325	Panopea Generosa	the United States	2007/4/19	-	4548093	150	5036645	30	45.38	463537	4	203×	4610	4524	81	1	4	-/-
vp.326	Scylla	the Philippines	2007/4/29	-	4403380	150	5249831	41	45.11	493345	4	195×	4830	4749	76	1	4	-/-
vp.328	Fiddler Crab	Bangladesh	2007/4/29	-	5155604	150	5009191	53	45.34	452921	4	204×	4594	4490	99	1	4	-/-
vp.332	Black Border Abalone	Australia	2007/4/29	-	3733400	150	6447767	2936	43.95	4067	301	159×	5938	5815	118	2	3	-/+
vp.333	Scylla	the Philippines	2006/12/11	-	7815436	150	6158601	742	44.24	80505	21	166×	5805	5686	114	2	3	-/-
vp.336	Gymnothorax	Canada	2006/6/27	-	5021193	150	6153587	63	44.07	400886	7	166×	5985	5825	155	2	3	-/-
vp.338	Panopea Generosa	Norway	2006/8/18	-	5727063	150	5095725	65	45.27	344613	5	201×	4660	4578	77	1	4	-/-
vp.348	Salmo Salar	Norway	2006/7/17	-	3644093	150	4911384	72	45.4	117805	12	208×	4475	4389	82	1	3	-/-
vp.351	Abalone	China	2006/6/27	-	6137768	150	5263724	48	45.25	543631	4	195×	4903	4823	76	1	3	-/-
vp.352	Hairtail	China	2005/12/21	-	6510038	150	5073900	55	45.33	401054	4	202×	4638	4556	77	1	4	-/-

vp.354	Panopea Generosa	Canada	2006/2/9	-	4569266	150	4968385	25	45.4	1240722	2	206×	4564	4466	94	1	3	-/-
vp.355	Grouper	China	2006/7/17	-	3961040	150	5019237	78	45.38	196992	8	204×	4599	4498	96	1	4	-/-
vp.357	Hairtail	China	2006/7/11	-	8547918	150	4958685	27	45.4	465621	4	207×	4535	4446	84	1	4	-/-
vp.360	Abalone	Burma	2005/12/21	-	4991418	150	5083464	43	45.33	400189	5	201×	4670	4579	86	1	4	-/-
vp.362	Acharax	China	2005/12/21	-	6732306	150	5140108	57	45.2	454238	5	199×	4714	4631	78	1	4	-/-
vp.363	Salmo Salar	Norway	2006/2/13	-	8738410	150	5326987	46	45.28	517974	3	192×	4972	4877	90	2	3	-/-
vp.364	Hairtail	China	2006/5/25	-	5378500	150	5036104	76	45.27	375021	5	203×	4638	4540	92	1	5	-/-
vp.366	Abalone	China	2006/6/27	-	5707498	150	5014699	50	45.32	450687	5	204×	4594	4497	92	1	4	-/-
vp.368	Hairtail	China	2006/7/4	-	4750883	150	5116640	79	45.34	153526	11	200×	4674	4577	92	1	4	-/-
vp.369	Salmo Salar	Chile	2006/7/4	-	7464872	150	6146878	70	44.02	446837	6	167×	5986	5843	137	2	4	-/-
vp.372	Salmo Salar	Chile	2006/7/11	-	3830626	150	5138621	49	45.18	450801	4	199×	4734	4648	82	1	3	-/-
vp.374	Oratosquilla Oratoria	China	2006/7/11	-	7306866	150	5232487	52	45.24	402857	4	196×	4790	4701	84	1	4	-/-
vp.380	Oratosquilla Oratoria	Thailand	2006/7/15	-	5298323	150	5314330	99	45.18	157217	9	193×	4924	4825	93	1	5	-/-
vp.383	Hairtail	China	2006/7/17	-	6848438	150	5306609	49	45.1	515007	5	193×	4889	4801	84	1	3	-/-
vp.390	Salmo Salar	Chile	2006/8/25	-	7076284	150	5059882	61	45.33	402175	5	202×	4642	4550	88	1	3	-/-
vp.391	Panopea Generosa	Canada	2006/8/18	-	8547918	150	5009725	37	45.33	366375	4	204×	4621	4541	76	1	3	-/-
vp.392	Dungeness Crab	Canada	2006/8/25	-	7550218	150	4995794	42	45.41	445779	4	205×	4599	4506	89	1	3	-/-
vp.393	Dungeness Crab	Canada	2006/8/25	-	8563628	150	6030296	47	44.01	395831	4	170×	5825	5706	113	2	4	-/-
vp.396	Hairtail	China	2007/9/14	-	6080350	150	5004594	44	45.26	451971	4	205×	4579	4485	90	1	3	-/-
vp.397	Cuttlefish	China	2007/9/14	-	4325110	150	5245812	69	45.2	135793	12	195×	4835	4742	89	1	3	-/-
vp.398	Hairtail	China	2007/9/14	-	5827344	150	4932268	36	45.34	695073	3	208×	4530	4432	94	1	3	-/-
vp.399	Dungeness Crab	Canada	2007/9/13	-	5122876	150	5023725	75	45.26	194788	9	204×	4649	4565	79	1	4	-/-
vp.401	Dungeness Crab	Canada	2007/9/14	-	4617976	150	5854150	91	44.1	114670	12	175×	5657	5500	151	2	4	-/-
vp.402	Dungeness Crab	Canada	2007/9/14	-	8426478	150	5074739	55	45.3	517149	4	202×	4617	4533	80	1	3	-/-
vp.404	Homarus	the United	2007/9/14	-	5298690	150	5941449	80	44.08	177253	10	172×	5721	5586	129	2	4	-/-

		States																
vp.405	Dungeness Crab	Canada	2007/9/14	-	5568516	150	5059695	79	45.26	163445	10	202×	4618	4533	81	1	3	-/-
vp.406	Dungeness Crab	Norway	2007/9/20	-	6194596	150	5058277	61	45.26	450926	4	202×	4616	4533	79	1	3	-/-
vp.408	Dungeness Crab	Norway	2007/9/20	-	7818622	150	5075934	36	45.35	561049	4	202×	4646	4556	86	1	3	-/-
vp.409	Brown Crab	UK	2007/9/20	-	9000426	150	4948056	63	45.37	442698	5	207×	4559	4473	82	1	3	-/-
vp.410	Dungeness Crab	Canada	2007/9/20	-	4410603	150	5041633	72	45.27	128496	11	203×	4677	4590	83	1	3	-/-
vp.411	Dungeness Crab	Canada	2007/9/20	-	8668748	150	5061398	63	45.33	402181	5	202×	4648	4556	88	1	3	-/-
vp.413	Dungeness Crab	the United States	2007/9/20	-	4781726	150	5219998	72	45.25	167256	11	196×	4773	4700	65	1	7	-/-
vp.414	Grouper	China	2007/9/20	-	5283366	150	5416075	176	45.22	232949	8	189×	5080	4991	85	1	3	-/-
vp.415	Panulirus Ornatus	Vietnam	2007/9/27	-	6077210	150	5033014	29	45.26	548917	3	203×	4583	4490	89	1	3	-/-
vp.416	Scylla	China	2007/9/27	-	4102823	150	6123775	67	44.51	228631	7	167×	5915	5788	121	2	4	-/-
vp.417	Red Spotted Crab	China	2007/9/27	-	4407643	150	5108146	64	45.3	194275	8	200×	4706	4614	88	1	3	-/-
vp.418	Dungeness Crab	Canada	2007/10/15	-	4991418	150	5004222	68	45.33	138834	11	205×	4609	4530	75	1	3	-/-
vp.419	Cray	China	2007/10/15	-	8738410	150	4738165	78	45.46	124114	12	216×	4309	4239	65	1	4	-/-
vp.421	Black Crab	India	2007/10/15	-	8104016	150	5196957	78	45.31	253016	7	197×	4790	4689	97	1	3	-/-
vp.423	Black Crab	India	2007/10/15	-	3830626	150	4977275	43	45.36	539352	4	206×	4540	4455	80	1	4	-/-
vp.424	Dungeness Crab	Canada	2007/10/15	-	5863948	150	5047466	65	45.32	309577	6	203×	4649	4552	92	1	4	-/-
vp.425	Cancer Pagurus	UK	2007/10/15	-	3963992	150	5830012	3077	44.83	3216	399	176×	5036	4960	72	1	3	-/-
vp.426	Black Crab	the Philippines	2007/10/15	-	4189266	150	5259642	114	45.32	134564	13	195×	4884	4791	88	1	4	-/-
vp.428	Black Crab	Thailand	2007/10/15	-	4868042	150	5009868	26	45.41	457037	4	204×	4585	4496	84	1	4	-/-
vp.429	Panopea Generosa	Canada	2007/10/26	-	4982940	150	5096131	321	45.28	29147	57	201×	4666	4585	75	1	5	-/-
vp.433	Lobster	Australia	2007/10/26	-	4673012	150	4935426	30	45.43	511076	4	207×	4482	4398	78	1	5	-/-
vp.442	Oyster	France	2007/11/17	-	3718560	150	4933678	72	45.4	136210	11	208×	4502	4421	76	1	4	-/-

vp.445	Lobster	Canada	2007/12/29	-	4159480	150	4808813	67	45.49	123528	12	213×	4383	4290	89	1	3	-/-
vp.446	Clam	China	2007/12/29	-	4839036	150	5345565	98	44.91	197455	10	192×	4997	4912	81	1	3	-/-
vp.448	Squid	China	2008/1/25	-	7953540	150	5265265	87	45.24	186609	10	194×	4828	4744	80	1	3	-/-
vp.449	Bass	China	2008/1/25	-	3887756	150	5160407	92	45.16	157572	11	198×	4800	4707	89	1	3	-/-
vp.451	Lobster	Australia	2008/1/25	-	4617153	150	5050474	42	45.3	387429	5	203×	4658	4568	86	1	3	-/-
vp.452	Frozen Hairtail	China	2008/1/25	-	7770836	150	5039536	35	45.29	407585	4	203×	4609	4520	85	1	3	-/-
vp.454	Frozen Croaker	China	2008/1/25	-	3815440	150	5029347	70	45.22	143072	12	204×	4622	4520	97	1	4	-/-
vp.458	Frozen Sole	China	2008/2/2	-	4030183	150	4941822	51	45.31	404210	5	207×	4564	4477	83	1	3	-/-
vp.460	Bass	China	2008/4/11	-	8134534	150	5070222	54	45.23	543635	4	202×	4729	4631	93	1	4	-/-
vp.468	Panopea Generosa	China	2008/4/11	-	2619166	150	5162278	67	45.21	163127	9	198×	4750	4666	80	1	3	-/-
vp.469	Clam	China	2008/4/11	-	3590736	150	5071745	84	45.32	161619	9	202×	4646	4549	93	1	3	-/-
vp.470	Croaker	China	2008/4/11	-	7550218	150	5064349	60	45.23	460513	4	202×	4722	4624	94	1	3	-/-
vp.471	Crocea	China	2008/4/11	-	4334236	150	4942438	59	45.38	460017	5	207×	4508	4425	78	1	4	-/-
vp.473	Alepocephalus Bicolor	China	2008/4/11	-	5541026	150	5067992	59	45.29	198504	6	202×	4675	4595	75	1	4	-/-
vp.475	Salmo Salar	Norway	2008/5/8	-	4824286	150	5101531	33	45.34	459606	4	201×	4664	4574	85	1	4	-/-
vp.477	Bass	China	2008/5/8	-	6623238	150	4996904	58	45.37	589109	3	205×	4599	4507	87	1	4	-/-
vp.482	Acharax	China	2008/5/13	-	4325110	150	4936349	69	45.29	297796	7	207×	4515	4427	84	1	3	-/-
vp.483	Salmo Salar	Norway	2008/5/13	-	5008790	150	5998180	80	44.05	161924	12	171×	5808	5649	153	2	4	-/-
vp.486	Gayu	China	2008/6/6	-	3990526	150	5250428	104	45.29	123324	13	195×	4878	4784	90	1	3	-/-
vp.489	Salmo Salar	Norway	2008/6/11	-	5035236	150	5052390	37	45.3	375158	6	203×	4598	4508	86	1	3	-/-
vp.492	Fish	China	2008/6/17	-	4548093	150	5262030	67	45.31	231764	7	195×	4887	4789	93	1	4	-/-
vp.493	Philippine Lobster	the Philippines	2008/6/20	-	5303523	150	5034237	65	45.18	733669	3	203×	4623	4510	108	1	4	-/-
vp.496	Oyster	France	2008/7/1	-	4327210	150	5105876	56	45.29	264366	7	201×	4726	4633	88	1	4	-/-

vp.498	Croaker	China	2008/7/4	-	4711513	150	4873710	38	45.37	453548	4	210×	4480	4393	83	1	3	-/-
vp.499	Dungeness Crab	the United States	2008/7/9	-	3699626	150	5080041	53	45.28	349746	6	202×	4627	4539	83	1	4	-/-
vp.500	Panopea Generosa	Canada	2008/7/9	-	6137768	150	5205127	59	45.31	284196	6	197×	4824	4728	91	1	4	-/-
vp.501	Sturgeon	China	2008/7/17	-	2619166	150	5226389	63	45.21	213405	9	196×	4773	4682	87	1	3	-/-
vp.502	Salmo Salar	Chile	2008/7/17	-	7306866	150	4975440	54	45.36	189433	9	206×	4535	4450	80	1	4	-/-
vp.505	Cray	Iceland	2008/7/26	-	3537810	150	4803866	168	44.57	51039	25	213×	4586	4486	94	2	4	-/-
vp.506	Phalacrocorax Carbo	China	2008/7/26	-	7154860	150	5044448	68	45.32	401923	5	203×	4650	4557	89	1	3	-/-
vp.507	Croaker	China	2008/7/29	-	3693506	150	5045513	86	45.31	124692	13	203×	4599	4515	79	1	4	-/-
vp.508	Crocea	China	2008/7/30	-	7121152	150	5176656	51	45.1	447445	4	198×	4786	4699	82	1	4	-/-
vp.509	Cuttlefish	China	2008/7/30	-	7426670	150	5177164	50	45.1	447445	4	198×	4792	4701	86	1	4	-/-
vp.511	Phalacrocorax Carbo	China	2008/7/30	-	4354313	150	5177691	53	45.1	273934	5	198×	4784	4696	83	1	4	-/-
vp.513	Cuttlefish	China	2008/8/27	-	10098026	150	5020303	53	45.34	443237	4	204×	4611	4512	93	1	5	-/-
vp.514	Oyster	New Zealand	2008/8/27	-	3881846	150	5022107	108	45.22	99230	16	204×	4595	4514	76	1	4	-/-
vp.515	Panopea Generosa	Canada	2008/8/27	-	4253306	150	4931595	50	45.4	238365	7	208×	4511	4412	95	1	3	-/-
vp.524	Leucothoidae	Ecuador	2008/9/12	-	3784213	150	4776647	42	45.4	377249	5	214×	4368	4280	84	1	3	-/-
vp.527	Hypophthalmichthys Molitrix	China	2008/9/23	-	7937460	150	5074292	33	45.25	583518	3	202×	4640	4552	84	1	3	-/-
vp.533	Dungeness Crab	the United States	2008/9/24	-	4981646	150	5111314	37	45.28	410493	5	200×	4683	4588	90	1	4	-/-
vp.535	Cynoglossus Robustus	China	2008/9/24	-	4041466	150	5266642	86	45.13	413516	6	194×	4833	4740	89	1	3	-/-
vp.539	Carp	China	2008/10/7	-	6325416	150	5119034	41	45.3	404560	5	200×	4717	4631	82	1	3	-/-
vp.551	Salmo Salar	Norway	2008/10/22	-	6238216	150	5022294	47	45.21	471146	5	204×	4576	4486	85	1	4	-/-
vp.553	Pandalus Borealis	Canada	2008/10/22	-	8065214	150	5011866	40	45.3	1050944	2	204×	4580	4500	75	1	4	-/-

vp.554	Salmo Salar	Norway	2008/10/22	-	5210116	150	6078390	134	44.08	124037	13	168×	5890	5740	143	2	5	-/-
vp.557	Scylla	Burma	2008/10/22	-	5160396	150	5255951	92	45.33	316968	7	195×	4883	4791	87	1	4	-/-
vp.561	Black Crab	Burma	2008/11/4	-	6228712	150	4954366	51	45.41	470897	5	207×	4555	4459	92	1	3	-/-
vp.562	Lobster	Canada	2008/11/4	-	6543746	150	5070424	28	45.3	683951	3	202×	4641	4548	88	1	4	-/-
vp.566	Salmo Salar	Norway	2008/11/4	-	8563628	150	4968979	40	45.39	750329	2	206×	4552	4468	80	1	3	-/-
vp.568	Cray	Finland	2008/11/21	-	4298433	150	6134547	676	44.1	27855	57	167×	5823	5696	124	2	1	-/-
vp.569	Cray	Finland	2008/11/21	-	3629490	150	4974953	51	45.41	368742	6	206×	4568	4482	82	1	3	-/-
vp.571	Salmo Salar	Norway	2008/12/18	-	6552996	150	5147966	347	45.28	77614	18	199×	4690	4613	73	1	3	-/-
vp.575	Panopea Generosa	Canada	2009/3/24	-	3531130	150	5123483	133	45.19	169518	10	200×	4791	4707	80	1	3	-/-
vp.581	Abalone	Indonesia	2009/5/19	-	5969292	150	5038330	37	45.28	547665	3	203×	4637	4552	80	1	4	-/-
vp.584	Scylla	Burma	2009/5/19	-	4322800	150	5245277	89	45.47	313973	5	195×	4803	4718	81	1	3	-/-
vp.586	Panopea Generosa	Canada	2009/5/19	-	7237082	150	5290657	95	45.28	231846	7	194×	4873	4780	88	1	4	-/-
vp.598	Hairtail	China	2009/8/14	-	5707498	150	4929760	34	45.38	545832	4	208×	4498	4423	70	1	4	-/-
vp.599	Crap	China	2009/8/17	-	6813412	150	4981166	44	45.32	451214	4	206×	4577	4490	82	1	4	-/-
vp.609	Pomfret	China	2009/10/23	-	3901430	150	5234566	89	45.25	179611	11	196×	4792	4706	82	1	3	-/-
vp.610	Croaker	China	2009/10/29	-	6693152	150	5022212	27	45.4	534777	3	204×	4597	4513	79	1	4	-/-
vp.611	Salmon	Canada	2009/12/18	-	3694730	150	5138273	84	45.22	248603	6	199×	4801	4716	80	1	4	-/-
vp.618	Gadus	Iceland	2010/5/13	-	3725100	150	4959548	57	45.4	265612	7	206×	4545	4458	82	1	4	-/-
vp.623	Wild Dalian Abalone	China	2010/7/15	-	3486816	150	6266839	1048	44.02	17627	97	163×	5985	5869	110	2	4	-/-
vp.624	Pomfret	China	2010/7/15	-	11790718	150	5260019	61	45.31	404824	5	195×	4862	4770	87	1	4	-/-
vp.626	Sardine	China	2010/7/15	-	4628090	150	5098289	55	45.34	462140	5	201×	4691	4597	90	1	3	-/-
vp.631	Cyprinidae	China	2010/7/15	-	5830166	150	4972770	35	45.39	414796	3	206×	4519	4436	79	1	3	-/-
vp.632	Scallops	China	2010/7/15	-	3443793	150	5027348	82	45.34	137170	9	204×	4653	4570	79	1	3	-/-
vp.641	Scallop	China	2010/7/20	-	3705986	150	5447869	2533	44.89	3742	362	188×	4716	4644	70	1	2	-/-

vp.650	Bass	China	2010/10/20	-	5179343	150	5128735	52	45.2	386502	6	200×	4722	4630	87	1	4	-/+

Table S3 Annotation summary of 241 *V. parahaemolyticus* strains.

Strains	CDS	tRNA	tmRNA	rRNA	Hypothetical protein	Function proteins
vp.301	4548	87	1	3	1519	3082
vp.302	4652	86	1	4	1631	3169
vp.303	4772	90	1	4	1722	2800
vp.304	4441	82	1	4	1459	3158
vp.305	5592	119	2	4	2435	2644
vp.306	5731	128	2	5	2556	3218
vp.307	5606	134	2	4	2446	2232
vp.308	4642	92	1	3	1614	3051
vp.310	4499	79	1	4	1507	3067
vp.311	4864	89	1	3	1791	2776
vp.312	4628	78	1	4	1596	3019
vp.313	4757	93	1	4	1744	4202
vp.314	5600	130	2	4	2403	2455
vp.315	4783	77	1	4	1735	3001
vp.317	4429	86	1	3	1434	4398
vp.318	4585	85	1	4	1575	2954
vp.319	4550	86	1	3	1529	3034
vp.320	4403	92	1	4	1411	3154
vp.321	4533	87	1	4	1545	3128
vp.322	4786	90	1	3	1722	2839
vp.323	5252	105	2	4	2125	2545
vp.324	4488	90	1	3	1495	3289
vp.325	4524	81	1	4	1498	3012
vp.326	4749	76	1	4	1725	3075
vp.327	4595	73	1	3	1606	2916
vp.328	4490	99	1	4	1485	3301
vp.330	4396	83	1	4	1404	3356
vp.331	5726	107	2	3	2517	2220
vp.332	5815	118	2	3	2971	1513
vp.333	5686	114	2	3	2540	2091
vp.334	4383	84	1	3	1402	3232
vp.335	4516	96	1	4	1507	3032
vp.336	5825	155	2	3	2612	2062
vp.337	5703	124	2	3	2492	2185
vp.338	4578	77	1	4	1586	4150
vp.339	4771	83	1	3	1732	3149
vp.340	5732	125	2	3	2528	2287

vp.342	4454	70	1	4	1462	3174
vp.344	5768	122	2	5	2608	2005
vp.345	5875	128	2	3	2675	3222
vp.347	4599	75	1	3	1586	3104
vp.348	4389	82	1	3	1393	3246
vp.349	5716	133	2	3	2518	3183
vp.350	4456	78	1	3	1466	3032
vp.351	4823	76	1	3	1774	2920
vp.352	4556	77	1	4	1540	3092
vp.353	4559	81	1	3	1545	3127
vp.354	4466	94	1	3	1471	3008
vp.355	4498	96	1	4	1484	3160
vp.356	6010	116	1	6	2333	2176
vp.357	4446	84	1	4	1446	2993
vp.358	4564	82	1	3	1539	2958
vp.360	4579	86	1	4	1568	2959
vp.362	4631	78	1	4	1601	2933
vp.363	4877	90	2	3	1815	2699
vp.364	4540	92	1	5	1521	3085
vp.366	4497	92	1	4	1493	3070
vp.367	4735	89	1	3	1681	2790
vp.368	4577	92	1	4	1550	2758
vp.369	5843	137	2	4	2639	2253
vp.371	5614	155	2	3	2446	2339
vp.372	4648	82	1	3	1629	3078
vp.374	4701	84	1	4	1650	2910
vp.375	4707	88	1	4	1645	2893
vp.376	4538	93	1	4	1539	3111
vp.377	4644	88	1	3	1618	4220
vp.378	5583	129	2	3	2413	3449
vp.380	4825	93	1	5	1810	4044
vp.381	5706	127	2	3	2526	3607
vp.382	4515	94	1	3	1518	4199
vp.383	4801	84	1	3	1722	4116
vp.384	4982	92	1	4	1956	2956
vp.385	4607	91	1	4	1585	2927
vp.386	4464	91	1	4	1485	4232
vp.390	4550	88	1	3	1526	3068
vp.391	4541	76	1	3	1553	3063
vp.392	4506	89	1	3	1503	4534
vp.393	5706	113	2	4	2520	2163
vp.394	4418	87	1	4	1430	3112

vp.395	4426	92	1	3	1465	3287
vp.396	4485	90	1	3	1468	3253
vp.397	4742	89	1	3	1672	2917
vp.398	4432	94	1	3	1431	3296
vp.399	4565	79	1	4	1566	3123
vp.400	4688	93	1	4	1648	3289
vp.401	5500	151	2	4	2360	2468
vp.402	4533	80	1	3	1504	4504
vp.403	4510	86	1	4	1494	3171
vp.404	5586	129	2	4	2414	2289
vp.405	4533	81	1	3	1545	3219
vp.406	4533	79	1	3	1546	3104
vp.408	4556	86	1	3	1556	4061
vp.409	4473	82	1	3	1489	3150
vp.410	4590	83	1	3	1617	3126
vp.411	4556	88	1	3	1532	3335
vp.412	4566	94	1	4	1551	2977
vp.413	4700	65	1	7	1627	4119
vp.414	4991	85	1	3	1903	2835
vp.415	4490	89	1	3	1447	3136
vp.416	5788	121	2	4	2573	2384
vp.417	4614	88	1	3	1592	3119
vp.418	4530	75	1	3	1542	3313
vp.419	4239	65	1	4	1376	3489
vp.420	4684	96	1	4	1667	2852
vp.421	4689	97	1	3	1653	3022
vp.423	4455	80	1	4	1454	3186
vp.424	4552	92	1	4	1551	2949
vp.425	4960	72	1	3	2332	2293
vp.426	4791	88	1	4	1741	3139
vp.427	4611	91	1	4	1603	3760
vp.428	4496	84	1	4	1502	3080
vp.429	4585	75	1	5	1592	3018
vp.430	4578	83	1	3	1572	3258
vp.431	4665	94	1	4	1635	2959
vp.432	4556	89	1	4	1533	4405
vp.433	4398	78	1	5	1405	4400
vp.435	4348	87	1	3	1399	4586
vp.437	4412	81	1	3	1440	3220
vp.438	4415	82	1	3	1441	3293
vp.439	4439	84	1	3	1459	3016
vp.441	4518	83	1	4	1516	3387

vp.442	4421	76	1	4	1440	3198
vp.443	4573	96	1	3	1552	3012
vp.444	4444	86	1	3	1464	3135
vp.445	4290	89	1	3	1333	3202
vp.446	4912	81	1	3	1879	3219
vp.448	4744	80	1	3	1684	2986
vp.449	4707	89	1	3	1670	3044
vp.450	4400	92	1	5	1405	3567
vp.451	4568	86	1	3	1659	2979
vp.452	4520	85	1	3	1509	3085
vp.453	4797	80	1	3	1725	2949
vp.454	4520	97	1	4	1496	4490
vp.455	4472	84	1	4	1463	3271
vp.456	4402	73	1	3	1413	3377
vp.458	4477	83	1	3	1578	3346
vp.459	4616	74	1	3	1599	3290
vp.460	4631	93	1	4	1619	3082
vp.462	4729	81	1	4	1713	2929
vp.467	4716	80	1	3	1695	2926
vp.468	4666	80	1	3	1632	2967
vp.469	4549	93	1	3	1559	4266
vp.470	4624	94	1	3	1612	2967
vp.471	4425	78	1	4	1423	3412
vp.472	4595	91	1	3	1565	2965
vp.473	4595	75	1	4	1603	3046
vp.474	4690	90	1	3	1618	4039
vp.475	4574	85	1	4	1537	3080
vp.476	4590	79	1	4	1564	4157
vp.477	4507	87	1	4	1509	3109
vp.478	4593	79	1	4	1568	3048
vp.481	4219	85	1	3	1332	3483
vp.482	4427	84	1	3	1507	3139
vp.483	5649	153	2	4	2477	2082
vp.484	4471	87	1	4	1484	3193
vp.485	4483	77	1	4	1494	3154
vp.486	4784	90	1	3	1723	3050
vp.487	4543	86	1	4	1544	3536
vp.489	4508	86	1	3	1505	3078
vp.490	4485	85	1	3	1488	4427
vp.491	4485	78	1	3	1487	3219
vp.492	4789	93	1	4	1725	2884
vp.493	4510	108	1	4	1486	2823

vp.496	4633	88	1	4	1624	3166
vp.498	4393	83	1	3	1416	3124
vp.499	4539	83	1	4	1509	3140
vp.500	4728	91	1	4	1677	3359
vp.501	4682	87	1	3	1634	3250
vp.502	4450	80	1	4	1451	3134
vp.503	4563	71	1	4	1529	3137
vp.504	5582	113	2	4	2420	2062
vp.505	4486	94	2	4	1817	2685
vp.506	4557	89	1	3	1545	2838
vp.507	4515	79	1	4	1519	3478
vp.508	4699	82	1	4	1707	3121
vp.509	4701	86	1	4	1710	3090
vp.510	5466	145	2	4	2385	2273
vp.511	4696	83	1	4	1705	2904
vp.512	5807	133	2	4	2604	2018
vp.513	4512	93	1	5	1527	3037
vp.514	4514	76	1	4	1520	3209
vp.515	4412	95	1	3	1431	3319
vp.524	4280	84	1	3	1374	3272
vp.525	4814	73	1	4	1760	2962
vp.526	4666	89	1	4	1625	2883
vp.527	4552	84	1	3	1511	3164
vp.528	4642	91	1	3	1626	3038
vp.533	4588	90	1	4	1535	3064
vp.535	4740	89	1	3	1651	2864
vp.536	4694	77	1	4	1684	4124
vp.537	4429	76	1	3	1451	3427
vp.538	4850	82	1	4	1709	2889
vp.539	4631	82	1	3	1601	3286
vp.541	4601	84	1	3	1615	3008
vp.551	4486	85	1	4	1496	3230
vp.553	4500	75	1	4	1488	2992
vp.554	5740	143	2	5	2566	2061
vp.555	4468	90	1	4	1478	3346
vp.557	4791	87	1	4	1740	3033
vp.558	4435	74	1	3	1429	3106
vp.560	4432	78	1	3	1454	3132
vp.561	4459	92	1	3	1468	3182
vp.562	4548	88	1	4	1499	3100
vp.563	4533	94	1	3	1490	3296
vp.566	4468	80	1	3	1472	3320

vp.568	5696	124	2	1	2555	2229
vp.569	4482	82	1	3	1484	3127
vp.571	4613	73	1	3	1615	2980
vp.573	4538	90	1	3	1547	2964
vp.574	4819	88	1	4	1734	2634
vp.575	4707	80	1	3	1722	2918
vp.577	4513	76	1	4	1516	3167
vp.579	4537	74	1	4	1509	3324
vp.581	4552	80	1	4	1548	3169
vp.583	5902	130	2	3	2670	1906
vp.584	4718	81	1	3	1632	2948
vp.585	4588	90	1	4	1579	4311
vp.586	4780	88	1	4	1726	3157
vp.592	4424	83	1	4	1498	3057
vp.598	4423	70	1	4	1455	3186
vp.599	4490	82	1	4	1512	3040
vp.601	4580	85	1	4	1580	4243
vp.602	4468	70	1	3	1488	3080
vp.606	4538	73	1	3	1543	3147
vp.609	4706	82	1	3	1656	3135
vp.610	4513	79	1	4	1511	3126
vp.611	4716	80	1	4	1706	3097
vp.613	4635	87	1	4	1639	3234
vp.618	4458	82	1	4	1466	3032
vp.620	4445	90	1	3	1444	3133
vp.622	4650	97	1	4	1628	3164
vp.623	5869	110	2	4	2731	1866
vp.624	4770	87	1	4	1655	3146
vp.625	4612	104	1	4	1593	2952
vp.626	4597	90	1	3	1576	4409
vp.629	4438	79	1	4	1462	3400
vp.631	4436	79	1	3	1444	3247
vp.632	4570	79	1	3	1554	2965
vp.640	4507	77	1	4	1514	3139
vp.641	4644	70	1	2	2013	2703
vp.650	4630	87	1	4	1580	3142

Table S4 MLST locus and ST type of 241 *V. parahaemolyticus.*

**Strains**	**Locus**							**ST**
	dnaE	*gyrB*	*recA*	*dtdS*	*pntA*	*pyrC*	*tnaA*	
vp.303	121	4	226	29	82	375	132	**2950***
vp.305	95	34	211	242	74	89	70	1295
vp.306	96	475	242	407	23	382	24	1678
vp.311	121	4	226	29	82	375	132	**2950**
vp.314	11	75	64	50	26	7	50	409
vp.321	14	158	97	150	31	297	26	**2951**
vp.324	249	347	226	345	69	11	187	**2952**
vp.325	266	316	25	330	50	10	62	**2953**
vp.327	167	4	152	103	107	153	47	843
vp.330	35	188	146	164	26	3	1	352
vp.331	301	426	65	252	212	71	54	1213
vp.334	60	94	89	82	26	37	23	1027
vp.335	17	16	13	7	24	16	20	28
vp.337	31	115	22	12	3	91	68	141
vp.339	47	58	53	104	26	37	26	138
vp.340	301	426	65	252	212	71	54	1213
vp.342	47	58	53	19	50	37	26	162
vp.344	42	248	19	76	129	46	69	470
vp.345	242	16	332	13	4	369	26	1521
vp.347	34	29	2	33	25	26	17	57
vp.349	301	426	65	252	212	71	54	1213
vp.350	55	15	31	55	54	337	46	1167
vp.351	80	106	34	237	23	391	26	**2954**
vp.353	28	17	21	119	20	23	24	896
vp.356	301	426	65	252	212	71	54	1213
vp.357	69	92	69	114	225	91	24	1842
vp.358	111	5	241	34	20	171	24	1202
vp.360	51	4	77	84	60	8	22	**2955**
vp.363	34	481	93	169	26	39	81	**2956**
vp.366	156	199	39	19	2	11	26	**2957**
vp.367	330	88	113	242	18	99	23	1449
vp.371	23	29	10	7	14	24	2	43
vp.372	36	4	188	72	28	168	37	**2958**
vp.374	12	461	31	13	26	145	47	**2959**
vp.375	7	36	4	26	6	18	26	10
vp.376	3	159	86	171	61	175	126	437
vp.377	47	58	53	104	26	37	26	138

vp.378	301	426	65	252	212	71	54	1213
vp.381	301	426	65	252	212	71	54	1213
vp.382	179	232	62	27	125	5	26	461
vp.384	20	25	15	13	7	11	5	34
vp.385	20	7	75	27	24	7	15	1558
vp.386	35	352	151	47	26	325	1	1106
vp.391	31	106	135	74	37	173	54	**2960**
vp.392	28	401	70	236	4	46	23	**2961**
vp.393	326	236	4	26	6	18	26	**2962**
vp.394	10	69	27	76	46	65	29	235
vp.395	17	16	13	36	15	31	26	23
vp.397	10	388	239	74	165	89	217	2528
vp.400	86	117	35	19	26	92	15	322
vp.403	3	4	19	4	29	4	22	3
vp.409	167	2	109	293	28	10	105	**2963**
vp.412	7	230	99	378	6	370	1	1526
vp.418	31	106	135	74	37	173	54	**2960**
vp.419	205	385	218	243	18	182	57	**2964**
vp.420	183	396	172	27	23	182	57	1061
vp.421	121	4	226	29	82	375	132	**2950**
vp.423	3	58	81	191	70	180	176	**2965**
vp.424	42	151	98	12	88	117	26	**2966**
vp.427	183	396	172	27	23	182	57	1061
vp.430	62	69	57	61	46	65	24	107
vp.431	20	7	75	27	24	7	15	1558
vp.432	20	7	75	27	24	7	15	1558
vp.435	201	101	255	135	26	294	17	923
vp.437	201	101	255	135	26	294	17	923
vp.438	201	101	255	135	26	294	17	923
vp.439	201	101	255	135	26	294	17	923
vp.441	264	364	67	330	184	11	132	955
vp.443	76	88	31	13	53	45	13	165
vp.444	201	101	255	135	26	294	17	923
vp.448	19	74	61	69	48	11	26	**2967**
vp.450	195	263	187	75	23	198	26	520
vp.453	2	113	72	94	26	83	23	411
vp.454	98	4	30	19	77	10	82	1764
vp.455	97	127	101	29	78	99	47	194
vp.456	60	354	250	305	26	54	84	846
vp.459	31	106	135	74	37	173	168	643
vp.460	148	467	93	153	26	10	71	2578
vp.462	2	113	72	94	26	83	23	411

vp.467	12	146	117	124	28	10	54	243
vp.468	10	388	239	74	165	89	217	2528
vp.469	320	448	89	177	28	11	57	**2968**
vp.470	148	467	93	153	26	10	71	2578
vp.472	96	228	31	3	120	131	33	476
vp.474	5	84	115	74	84	26	84	234
vp.476	3	106	19	175	28	37	26	377
vp.478	3	106	19	175	28	37	26	377
vp.481	60	94	89	82	26	37	23	1027
vp.482	151	147	67	29	154	145	105	**2969**
vp.483	353	4	19	416	60	173	33	**2970**
vp.484	47	58	53	19	50	37	26	162
vp.485	47	58	53	19	50	37	26	162
vp.486	121	4	226	29	82	375	132	**2950**
vp.487	19	196	149	171	105	11	23	370
vp.490	10	1	62	95	50	85	26	124
vp.491	10	1	62	95	50	85	26	124
vp.492	121	4	226	29	82	375	132	**2950**
vp.500	121	4	226	29	82	375	132	**2950**
vp.501	212	25	11	69	18	45	26	**2971**
vp.502	3	58	81	191	70	180	176	**2965**
vp.503	71	257	183	29	132	26	24	507
vp.504	71	257	183	29	132	26	24	507
vp.505	175	113	204	230	26	35	51	**2972**
vp.506	44	130	87	110	26	37	63	**2973**
vp.507	28	15	31	55	18	58	46	1969
vp.510	358	484	355	314	23	392	2	1726
vp.512	85	13	67	1	35	88	67	130
vp.514	36	304	188	218	28	168	37	2220
vp.525	224	90	273	19	4	238	68	1060
vp.526	86	117	35	19	26	92	15	322
vp.528	20	25	15	13	7	3	5	324
vp.536	2	113	72	94	26	83	23	411
vp.537	98	4	112	107	77	97	204	916
vp.538	44	357	31	229	6	37	24	1354
vp.541	190	15	31	55	18	58	23	1353
vp.551	1	87	70	161	176	11	86	**2974**
vp.555	31	343	207	201	144	7	108	1018
vp.558	33	261	39	88	4	45	23	1305
vp.560	201	101	255	135	26	294	17	923
vp.561	121	295	25	19	31	90	24	**2977**
vp.563	12	180	81	19	21	11	73	363

vp.566	123	380	25	27	4	180	24	2791
vp.573	215	344	144	76	48	232	26	809
vp.574	117	13	97	69	26	190	23	1225
vp.577	169	226	3	50	23	8	33	1313
vp.579	12	130	86	91	4	102	23	1315
vp.583	51	4	218	84	60	8	33	1318
vp.585	42	343	89	13	61	188	50	1320
vp.592	60	197	31	18	106	150	26	1256
vp.598	205	385	218	243	18	182	57	**2964**
vp.601	69	92	69	114	54	71	24	212
vp.602	114	159	75	108	4	50	94	1326
vp.606	10	1	62	95	50	85	26	124
vp.611	2	113	72	94	26	83	62	**2975**
vp.613	49	323	25	29	1	39	17	1347
vp.618	123	380	25	27	4	180	24	2791
vp.620	70	175	31	81	49	74	62	373
vp.622	81	90	25	365	26	177	23	1331
vp.625	6	252	39	267	155	243	24	1334
vp.629	27	84	127	139	54	124	37	283
vp.640	95	34	211	242	74	45	70	1343
vp.650	123	100	127	315	66	7	31	**2976**

*** The bolded was the newly discovered STs in this study.**

Table S5 Information on the 9 mutation sites and their predicted protein-protein binding rates

SNP site	Amino acid site	Protein-protein binding prediction
A180G	P60P	10.20%
T552G	V184V	0.80%
G657T	*E219D*	33.30%
T858C	D286D	1.70%
C1062T	S354S	4.40%
A1137G	A379A	59.30%
T1179C	H393H	0.60%
**T259C**	*F87L*	53.40%
**A951T**	A317A	48.30%

***The bolded was the sites specific to clinical strains; the italics was the sites with non-synonymous mutations.**
